# PPIP5K2 Facilitates Proliferation and Metastasis of Non-Small Lung Cancer (NSCLC) through AKT Signaling Pathway

**DOI:** 10.3390/cancers16030590

**Published:** 2024-01-30

**Authors:** Qi Yang, Chenhui Cao, Binghuo Wu, Haochi Yang, Tian Tan, Dan Shang, Chuan Xu, Xiaoyi Huang

**Affiliations:** 1 Biotherapy Center, Harbin Medical University Cancer Hospital, Harbin 150001, China; yangqi97@126.com; 2 Sichuan Cancer Hospital & Institute, Sichuan Cancer Center, School of Medicine, University of Electronic Science and Technology of China, Chengdu 610054, China; caochenhui@uestc.edu.cn; 3 Sichuan Provincial Key Laboratory for Human Disease Gene Study and the Center for Medical Genetics, Department of Oncology & Cancer Institute, Sichuan Academy of Medical Sciences and Sichuan Provincial People’s Hospital, University of Electronic Science and Technology of China, Chengdu 610072, China; 4 School of Basic Medical Sciences, Chengdu University of Traditional Chinese Medicine, Chengdu 611137, China; 5 NHC Key Laboratory of Cell Transplantation, The First Affiliated Hospital of Harbin Medical University, Harbin 150001, China

**Keywords:** PPIP5K2, NSCLC, proliferation, EMT-dependent metastasis, AKT/mTOR pathway

## Abstract

**Simple Summary:**

Despite significant advancements in the detection and treatment of non-small lung cancer (NSCLC) over the last 20 years, the long-term benefits of treatment are limited due to the development of therapy resistance and distal metastasis. Therefore, the molecular mechanisms underlying NSCLC tumorigenesis must be identified to develop novel therapeutic targets to lessen malignancy and enhance patient outcomes. This study aimed to investigate how Diphosphoinositol Pentakisphosphate Kinase 2 (PPIP5K2) influenced the proliferation and metastasis of NSCLC by modulating AKT/mTOR signaling pathway using functional and mechanistic analyses. Our findings might contribute to the creation of innovative prognostic and therapeutic strategies for NSCLC patients.

**Abstract:**

Through facilitating DNA homologous recombination repair, PPIP5K2 has been proven to be essential for improving colorectal cancer survival in our previous research. However, its function in the tumorigenesis of NSCLC, the most common cancer and the primary cause of cancer-related death globally, is still unknown. Here, we initially discovered that PPIP5K2 had significant effects on proliferation of NSCLC cells through loss- and gain-of-function assays in vitro and in vivo. Moreover, PPIP5K2 is capable of regulating NSCLC cells metastasis in an EMT-dependent manner. In terms of mechanism exploration, we found that PPIP5K2 knockdown can significantly inhibit the phosphorylation of AKT/mTOR signaling pathway, whereas the overexpression of PPIP5K2 resulted in converse effects. By employing AKT signaling related agonists or antagonists, we further demonstrated that PPIP5K2 regulates NSCLC tumorigenesis partly via the AKT/mTOR pathway. In conclusion, PPIP5K2 plays a key oncogenic role in NSCLC by the activation of the AKT/mTOR signaling axis. It is anticipated that targeting PPIP5K2 might emerge as a viable therapeutic approach for NSCLC patients.

## 1. Introduction

Lung cancer is the leading cause of cancer-related death worldwide, accounting for the highest mortality rates among both men and women. Of these, NSCLC is the most common type, accounting for 80~85% [1,2]. Despite the substantial advances made in treating NSCLC with targeted therapies and immunotherapy with immune checkpoint inhibitors, on the basis of current clinical and experimental research data, patients ultimately develop disease progression due to acquired resistance [3,4]. Therefore, we still need to investigate a novel molecular mechanism for the development and progression of NSCLC and seek trustworthy therapeutic targets to improve the outcome of patients with NSCLC.

The PI3K/AKT/mTOR signaling is one of the most critical intracellular signaling pathways. Specifically, phosphatidylinositol-3,4,5-triphosphate (PIP3), a second-messenger molecule generated by the action of PI3K, activates the serine-threonine kinase AKT and regulates vital cellular functions, including quiescence, survival, cell cycle, and apoptosis in both healthy conditions and a variety of pathological diseases, including cancer [5,6]. Almost all solid tumors have been found to have hyperactivated PI3K/AKT/mTOR signaling, which is accountable for drug resistance [7,8]. Drugs that target this signaling, however, have not yet shown promising outcomes in the treatment of tumors [9]. As a result, research into the signaling molecules that control the PI3K/AKT/mTOR signaling is still necessary.

PPIP5K2 synthesize inositol pyrophosphates (PP-IPs), primarily by converting 5-diphosphoinositol pentakisphosphate (5-IP7) to 1,5-bisdiphos-phoinositol tetrakisphosphate (1,5-IP8) in vivo [10]. In our previous study, we confirmed that PPIP5K2 could be dephosphorylated, entered the nucleus after DNA damage, and participated in DNA homologous recombination repair to regulate the tumorigenesis of colorectal cancer [11]. Nevertheless, few relevant reports of PPIP5K2 in other cancer types, particularly NSCLC, have been found. Here, we report that PPIP5K2 inhibit the proliferation and EMT-dependent metastasis of NSCLC in vitro and in vivo. Importantly, we confirmed that PPIP5K2 significantly regulate the AKT/mTOR signaling pathway in NSCLC cells. We additionally demonstrated that PPIP5K2 partially controls NSCLC tumorigenesis through the AKT/mTOR signaling pathway via the application of AKT signaling-related agonists or antagonists. This suggests that PPIP5K2 may be used as a diagnostic and therapeutic target for NSCLC therapy.

## 2. Materials and Methods

Cell culture, The human NSCLC cell lines A549, NCI-H1299, NCI-H1975, and NCI-H1703 were all obtained from the American Type Culture Collection (ATCC). All NSCLC cell lines were cultured in RPMI-1640 (C22400500CP, Gibco, Waltham, MA, USA) with 10% FBS (F101-01, Vazyme, Nanjing, China). The human kidney HEK293T cell line was purchased from the ATCC and cultured in DMEM medium (12800082, Gibco, Waltham, MA, USA) with 10% FBS. Cells were cultured at 37 °C in an incubator (Esco Lifesciences, Changi, Singapore) with 5% CO_2_. We performed STR testing every six months and mycoplasma testing kits every month.

Plasmids and transfection, The PPIP5K2 overexpression and short-hairpin RNA (shRNA) were provided by Genecopoeia and transfected using jetPRIME^®^ (Polyplus-transfection, Illkirch-Graffenstaden, France). The overexpression plasmid we purchased did not contain a lentiviral vector, and it was modified on pLVX-Myc-MCS-3FLAG-PGK-Puro plasmid for subsequent experiments. The sequences of shRNA are as follows:

shA: gatccgGGAAATTATCGACATTTCTTCTCAAGAGGAAGAAA

TGTCGATAATTTCCttttttg.

shB: gatccgGGAAATATTGTAATGCGAGAATCAAGAGTTCTCGC

ATTACAATATTTCCttttttg.

Animal experiments, Male athymic nude mice, 4- to 5-week-old, were purchased from Huafukang Animal Centre, housed under standard conditions in the animal care facility at the Center of Animal Experiments of Sichuan Provincial People’s Hospital. Treated A549 cells (5 × 10^6^) were injected subcutaneously into the dorsal flanks of mice (*n* = 8/group), and tumor volume was measured every two days after tumor formation. After 6 weeks, mice were sacrificed, and tumors were excised, weighed, and then fixed with 4% paraformaldehyde.

Clinical samples, We collected surgical specimens (cancerous and paracancerous tissues) from 77 NSCLC patients at the Sichuan Provincial People’s Hospital affiliated with the University of Electronic Science and Technology. This study was approved to be conducted by Sichuan Academy of Medical Sciences, Sichuan Provincial People’s Hospital and University of Electronic Science and Technology, and informed consent was obtained from all patients. Ethical Review Approval Number: (Research) No. 332 of 2023. The tissues were placed in dry ice immediately after removal and then transferred to −80 °C for storage. The 77 pairs of tissue samples contained 72 adenocarcinomas and 5 squamous carcinomas.

RNA isolation and real-time quantitative PCR, The total RNA was extracted by the RNA extraction kit (RC112, Vazyme). RNA was reverse transcribed with a reverse transcription reagent (RK20433, ABclonal, Woburn, MA, USA) to obtain the desired cDNA. The qPCR experiment was conducted by using ChamQ Universal SYBR qPCR Master regents (Q712, Vazyme) following the manufacturer’s instructions. The relative expression levels of each gene were calculated using the 2^−ΔΔCt^ method and normalized to the levels of *gapdh*. The primer sequences are shown in Table 1.

Western blotting, A mixture of 1× RIPA lysis buffer with protease inhibitor cocktail was added to the cells, then lysed on ice for 30 min and centrifuged at 13,500 rpm for 15 min to obtain the supernatant. The protein concentration was determined by the BCA method. A total of 40 μg of proteins were separated with 10% SDS-PAGE gel and transferred to a PVDF membrane (10600023, Cytiva, Marlborough, MA, USA) and then blocked with 5% BSA. The primary antibody was incubated overnight at 4 °C, and the secondary antibody was incubated for 1 h at room temperature; after washing, the membrane was incubated with the ECL reagent (P10300, NCM biotech, Newport, RI, USA). The information on antibodies is shown in Table 2. Full-length blots/gels are presented in Supplementary Figure S1.

Transwell assay, The cells were starved for 12 h before the experiment began. After enzymatic digestion, the cells were washed twice with PBS and 5 × 10^4^ of A549, H1299, or H1975 cells, or 1 × 10^5^ H1703 cells suspended in 200 μL serum-free medium were added into the upper chamber. Concomitantly, 600 µL medium with 10% FBS was added to the lower chamber. The chambers were incubated at 37 °C for 24 h. Cells on the lower surface of the scrubbed membranes were fixed with methanol and stained with crystal violet.

Wound healing assay, Lines were drawn at the bottom of the 6-well plate beforehand and the digested and counted cells were plated. Once the cell density in the well plate increased to 90% in confluence, the medium was changed to serum-free medium for 6–12 h, and the cells were artificially slapped with the pipette tip to make the corresponding gaps. Then, the floating cells were washed off repeatedly with PBS, and pictures were taken to detect migration changes of cells at 0, 12, 24, and 48 h under the microscope.

CCK-8 assay, After washing and cell counting, 1 × 10^3^ NSCLC cells were cultured in a 96-well plate for 24 h. Then, 10 μL CCK-8 reagent and 90 μL serum-free 1640 medium were added to each well. The OD value at 450 nm was determined with a microplate reader 1 h later.

Cell colony formation assay, A total of 800 NSCLC cells (2 × 10^3^ cells when giving medicine) were placed in a 6-well plate and maintained in RPMI1640 containing 10% FBS for two weeks. When the cell clone has grown to an appropriate size, cells were fixed, stained with crystal violet, photographed, and counted.

Bioinformatics analysis, RNA-sequencing expression (level 3) profiles and corresponding clinical information for PPIP5K2 were downloaded from the TCGA dataset (https://portal.gdc.com, accessed on 27 December 2022). The current-release (V8) GTEx datasets were obtained from the GTEx data portal website (https://www.gtexportal.org/home/datasets, accessed on 27 December 2022). Statistical analyses were performed using R software v4.0.3 (R Foundation for Statistical Computing, Vienna, Austria). All microarray data were downloaded from the GEO database (http://www.ncbi.nih.gov/geo, accessed on 15 January 2024). The raw data were downloaded as MINiML files.

Statistical analysis, Statistical analyses were performed with GraphPad Prism v. 8 software by an unpaired two-tailed Student’s *t* test or by two-way ANOVA. All data are shown as mean ± SD. Each experiment was conducted with biological replicates and repeated no less than three times, with a value of *p* < 0.05 considered statistically significant.

## 3. Results

### 3.1. PPIP5K2 Promotes NSCLC Cell Proliferation In Vitro

**Figure 1:**
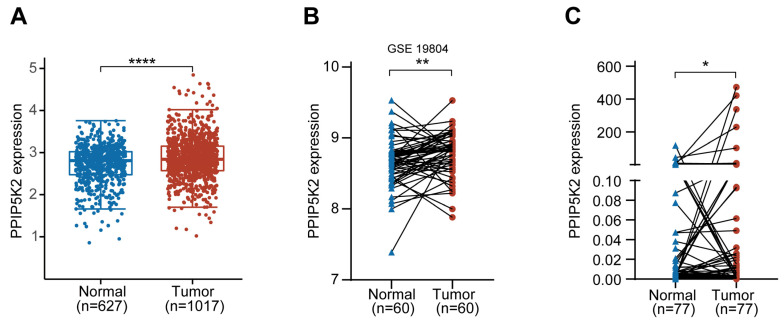
PPIP5K2 is highly expressed in NSCLC. (**A**) The distribution of PPIP5K2 gene expression in NSCLC and normal tissues in the TCGA database. (**B**) The expression distribution of PPIP5K2 gene in NSCLC tumor tissues and paired paraneoplastic tissues in the GEO database. (**C**) RT-qPCR analysis showing PPIP5K2 expression distribution cancer and paracancer tissues in 77 paired NSCLC patients. *p* values were determined using the Wilcox test in (**A**–**C**). * *p* < 0.05, ** *p* < 0.01, **** *p* < 0.0001.

It has been observed that PPIP5K2 possesses an important function in the tumorigenesis of a number of cancers, including ovarian and colorectal cancer, suggesting that PPIP5K2 is a potentially significant oncogene in malignancies [12,13,14]. Nevertheless, there are currently no reports on PPIP5K2 in lung cancer. We first performed a series of assays in the TCGA database and found that PPIP5K2 was highly expressed in NSCLC (Figure 1A). Moreover, we found that the expression level of PPIP5K2 in NSCLC cancer tissues was much higher than its paired paracancerous tissues in the GEO database (GSE19804) (Figure 1B). The findings derived from the database prompted us to ask for validation. We collected the cancer and paracancerous tissues from 77 NSCLC patients in the clinic and extracted the total RNA for subsequent RT-qPCR experiments, and the results were consistent with the interrogating readouts derived form GEO (Figure 1C).

**Figure 2:**
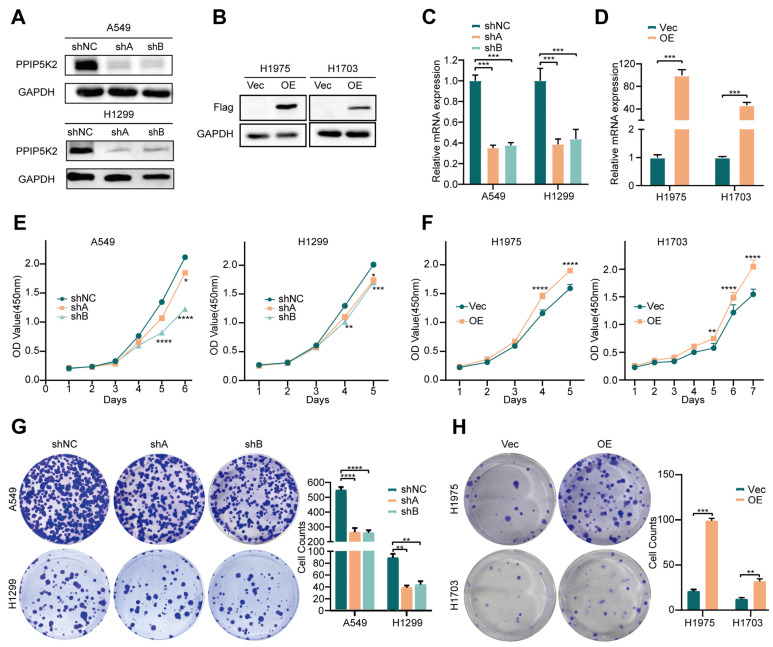
PPIP5K2 promotes NSCLC cell proliferation in vitro. (**A**,**B**) Western blot analysis showing the knockdown and overexpression efficiency of PPIP5K2. (**C**,**D**) RT-qPCR analysis showing the knockdown and overexpression efficiency of PPIP5K2. (**E**,**F**) CCK-8 assay showing the cell proliferation when PPIP5K2 changes. (**G**,**H**) Cell clone formation assay showing the cell proliferation when PPIP5K2 changes. Scale bar, 100 μm. All data represent the mean ± SD of three independent experiments. *p* values were determined using two-way ANOVA in (**C**–**H**). * *p* < 0.05, ** *p* < 0.01, *** *p* < 0.001, and **** *p* < 0.0001.

To study the biological functions of PPIP5K2 in NSCLC cells, we designed two short-hairpin RNAs targeting different regions of human PPIP5K2 mRNA and established PPIP5K2 stably knocked-down A549 and H1299 cells. Meanwhile, H1975 and H1703 were used for gain-of-function assays where PPIP5K2 was ectopically expressed using lentiviral-vector. Western blot and RT-qPCR were performed to confirm PPIP5K2 knockdown or overexpression efficiency in the constructed cell lines (Figure 2A–D). Next, we assessed how PPIP5K2 affected NSCLC cells proliferation. Upon PPIP5K2 depletion (shPPIP5K2), cell proliferation (Figure 2E), and colony-forming ability (Figure 2F) of A549 and H1299 cells were significantly reduced compared with the control cells. In contrast, we discovered that the rate of cellular proliferation and clone formation were significantly increased in cell lines overexpressing PPIP5K2 (Figure 2G,H). These findings imply that PPIP5K2 enhance the proliferation potential of NSCLC cells in vitro.

### 3.2. PPIP5K2 Possesses Oncogenic Capacities of NSCLC Cells In Vivo

**Figure 3:**
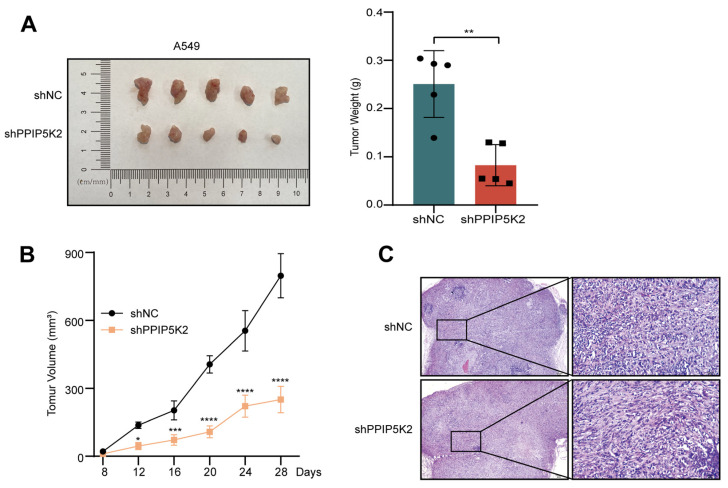
PPIP5K2 enhances NSCLC cells’ oncogenic capacities in vivo. (**A**) Representative images and weight of subcutaneous tumors in nude mice with injection of A549 cells. (**B**) The volume of subcutaneous tumors. (**C**) H&E staining of tumors from different groups. Scale bar, 100 μm. Data represent the mean ± SD in (**A**,**B**). *p* values were determined using Student’s *t* test in (**A**) and two-way ANOVA in (**B**). * *p* < 0.05, ** *p* < 0.01, *** *p* < 0.001, and **** *p* < 0.0001.

To deepen our understanding of this process, we established a xenograft tumor model by subcutaneously injecting control and shPPIP5K2 NSCLC cells into nude mice to investigate whether PPIP5K2 promote NSCLC cell proliferation in vivo. Following the injection of treated A549 cells to immune-deficient mice, it was shown that the tumor weight (Figure 3A) and volume (Figure 3B) of shPPIP5K2 cell-derived tumors were much smaller than those of control tumors. The results of the above experiment were also confirmed by H&E staining (Figure 3C). Combining all these data above, PPIP5K2 was proven to play an important role in the progression of NSCLC.

### 3.3. PPIP5K2 Is Essential for EMT-Dependent Cell Migration in NSCLC

**Figure 4:**
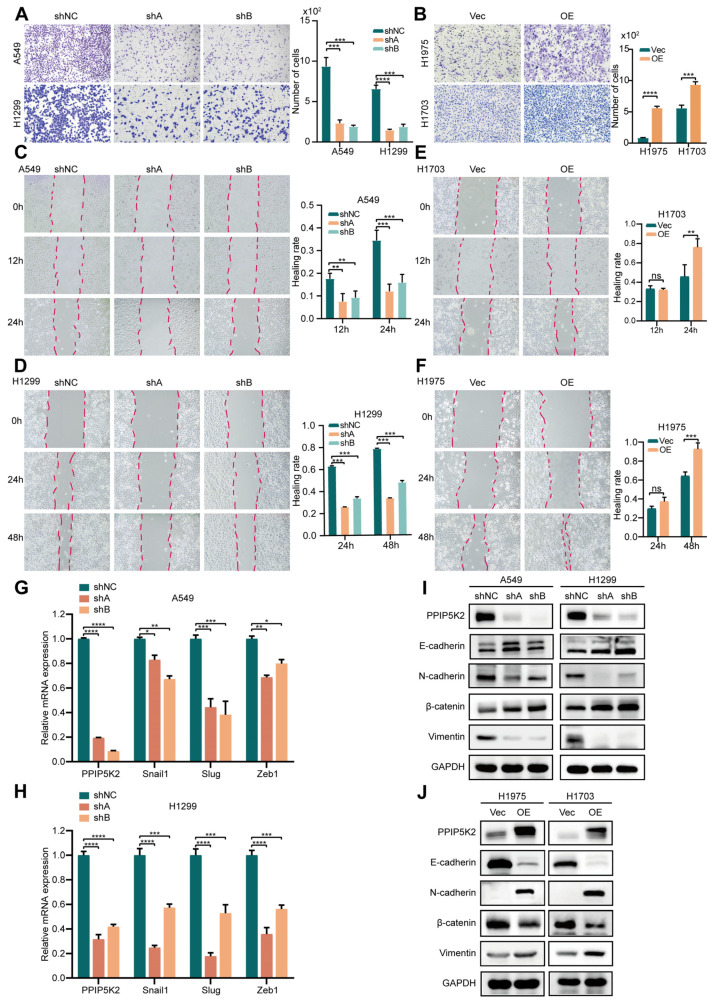
PPIP5K2 is essential for EMT-associated cell migration in NSCLC. (**A**,**B**) Transwell assay showing migration of knockdown and overexpression PPIP5K2 NSCLC cells. Scale bar, 100 μm. (**C**–**F**) Wound healing assay showing the migration of NSCLC cells after knockdown and overexpression PPIP5K2. Scale bar, 100 μm. (**G**,**H**) Representative qPCR analysis showing the levels of snail1, slug, and zeb1 when knockdown PPIP5K2 in NSCLC cells. (**I**,**J**) Western blot analysis of E-cadherin, N-cadherin, β-catenin, Vimentin expression in PPIP5K2 knockdown (A549, H1299) and overexpression (H1975, H1703) cell lines. Data are shown as the mean ± SD of three independent experiments. *p* values were determined using two-way ANOVA in (A-H). * *p*  <  0.05, ** *p*  <  0.01, *** *p*  <  0.001, and **** *p* < 0.0001, ns, nonsignificant.

There have not yet been any studies that investigate the role of PPIP5K2 in lung cancer metastasis. Here, PPIP5K2 was suggested to promote the metastatic potential of both PPIP5K2 knocked-down and overexpressed NSCLC cells (Figure 4A,B). Furthermore, the wound healing assays revealed that the migration of shPPIP5K2 cells was significantly slower compared to that of the control cells (Figure 4C,D). In the wound healing assays performed using PPIP5K2 overexpressed cells, no statistically significant changes were found at the first time point, but over time there was a significant statistical significance between the control group and the experimental group at the second evaluation (Figure 4E,F). These results suggest that PPIP5K2 increases the metastasis ability of NSCLC cells.

An important process by which tumor cells develop their migratory properties is considered to be EMT. EMT-inducing transcription factors, like Twist, Snail, Slug, and Zeb, are crucial mediators of cellular plasticity, thereby favoring progression to metastasis through different signaling cascades, including the AKT/mTOR, MAPK, and Wnt pathways [15]. Thus, we next investigated the role of PPIP5K2 in EMT. First, we examined a series of EMT-inducing transcription factors by RT-qPCR and found that the expression levels of Snail, Slug, and Zeb1 were reduced after knockdown of PPIP5K2, whereas PPIP5K2 overexpression enhanced the transcription of the markers (Figure 4G,H). Considering that EMT is characterized by a loss of epithelial markers, including E-cadherin and β-catenin, and an upregulation of mesenchymal markers, including N-cadherin and vimentin, the expression levels of these markers were further analyzed. PPIP5K2 silencing increased the expression of E-cadherin and β-catenin and, conversely, decreased the expression levels of vimentin and N-cadherin in A549 and H1299 cells (Figure 4I). In contrast, PPIP5K2 overexpression led to a decrease in E-cadherin and β-catenin expression in NSCLC cells, which was accompanied by an increase in N-cadherin and vimentin (Figure 4J). These results, taken together, suggested that PPIP5K2 was an essential regulatory protein implicated in EMT-dependent tumor metastasis.

### 3.4. PPIP5K2 Promotes the Proliferation and Metastasis of NSCLC Cells through the AKT/mTOR Pathway

**Figure 5:**
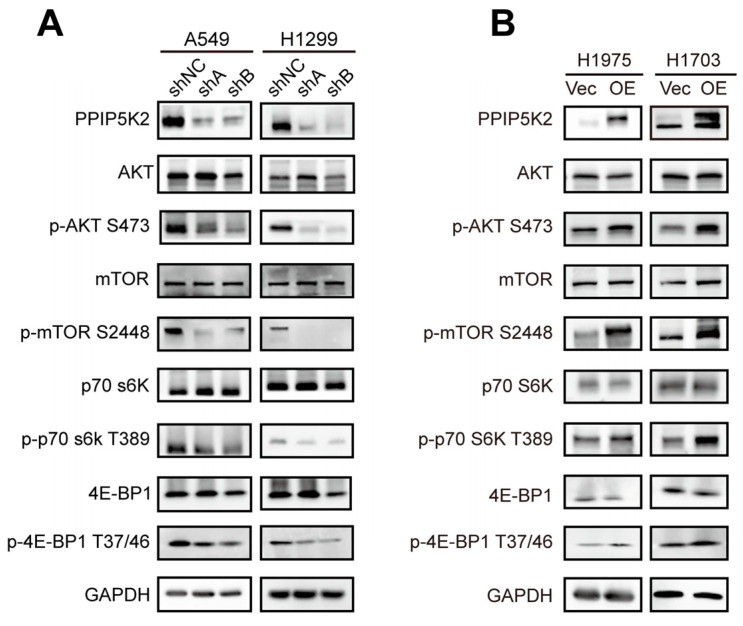
PPIP5K2 alterations could affect the AKT/mTOR pathway. (**A**,**B**) Western blot analysis of a series of AKT/mTOR pathway proteins expression in A549, H1299, H1975, and H1703 cell lines.

It has been demonstrated that the majority of oncogenic drivers, including EGFR and KRAS, activate the PI3K/AKT/mTOR pathway, which is essential for tumorigenesis and metastasis in NSCLC [16,17,18]. PI3K generates the PIP3 at the plasma membrane to recruit proteins that contain pleckstrin homology (PH) domains and 5-IP7, a soluble PIP3 analog, interferes with the binding of PIP3 to the proteins in the PH domain [19,20]. The inositol pyrophosphate 5-IP7, which could further be phosphorylated to 1,5-IP8 by PPIP5Ks, has been reported as a physiologic inhibitor of AKT [21]. Thus, kinases that target inositol pyrophosphates metabolism and IP7 storage can become novel therapeutic targets for the AKT signaling pathway. Nonetheless, the regulation of the AKT pathway by PPIP5K2 is still poorly understood. Here, we analyzed the expression of the key molecules in AKT/mTOR pathway, showing that the ratio of p-AKT/AKT, p-mTOR/mTOR, p-p70S6K/p70S6K, and p-4E-BP1/4E-BP1 significantly downregulated in PPIP5K2 silencing NSCLC cells, compared with control cells by western blot (Figure 5A). On the contrary, the phosphorylation levels of both AKT/mTOR and their downstream effectors were significantly increased in PPIP5K2 overexpressing NSCLC cells (Figure 5B). These findings suggested that PPIP5K2 is a new essential molecule involved in the AKT/mTOR pathway.

**Figure 6:**
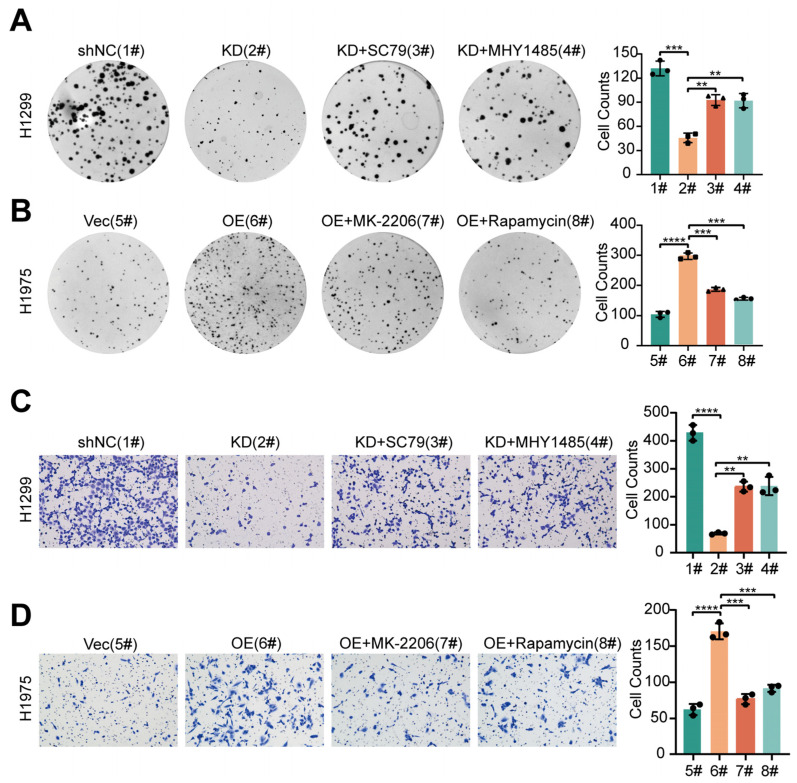
PPIP5K2 promotes proliferation and metastasis of NSCLC cells through AKT/mTOR pathway. (**A**,**B**) Colony formation assay showing the proliferation of H1299^PPIP5K2 knockdown^ and H1975^PPIP5K2 OE^ cell lines after treatment with SC79 (10 μm), MHY1485 (10 μm), MK-2206 (1 μm), and Rapamycin (1 μm). Scale bar, 100 μm. (**C**,**D**) Transwell assay showing the migration of H1299^PPIP5K2 knockdown^ and H1975^PPIP5K2 OE^ cell lines after treatment with SC79 (10 μm), MHY1485 (10 μm), MK-2206 (1 μm), and Rapamycin (1 μm). Scale bar, 100 μm. All data represent the mean ± SD of three independent experiments. *p* values were determined using Student’s *t* tests in (**A**–**D**). ** *p* < 0.01, *** *p* < 0.001, and **** *p* < 0.0001.

To determine the extent to which PPIP5K2 stimulates NSCLC cell proliferation and migration via the AKT/mTOR pathway, we applied the AKT activator (SC79) and mTOR activator (MHY1485) to perform rescue experiments in PPIP5K2 knocked-down NSCLC cells. We observed that the proliferative capacity of cells, which was originally inhibited by PPIP5K2 silencing, was partially restored by the addition of the agonists (Figure 6A). However, when we added AKT antagonist (MK2206) or mTOR antagonist (Rapamycin) to PPIP5K2 overexpressing cells, cell proliferation was fully recovered (Figure 6B). Comparably, we examined the effects of various AKT pathway agonists or antagonists on PPIP5K2-mediated lung cancer metastasis and discovered that a portion of the metastatic potential of PPIP5K2 knockdown cells was recovered by the addition of agonists, whereas the metastatic potential of PPIP5K2 overexpressing cells was completely restored by the addition of inhibitors (Figure 6C,D). The above experiments confirmed that the proliferative and metastatic function of PPIP5K2 on NSCLC is mainly, but not exclusively, mediated by the AKT/mTOR signaling pathway.

## 4. Discussion

In all eukaryotic cells, PP-IPs and their metabolizing enzymes (three isoforms of IP6Ks that add a β-phosphate at position 5 and two isoforms of PPIP5Ks that add a β-phosphate at position 1) regulate a variety of crucial cell biological activities, such as diet-induced obesity, type-2 diabetes, bacterial infection, cancer metastasis, and aging [10,22,23,24,25,26]. Naturally occurring human variants of PPIP5K2 have been associated with hearing loss and familial keratoconus [27,28]. In addition, PPIP5K2 has been reported to have a significant impact on the maintenance of hematopoietic stem cell activity [29] and tumorigenesis of colorectal cancer [11,13]. In this study, we investigated to what extent PPIP5K2 acted as a key oncogene in NSCLC and its underlying mechanisms. The pathological origins of the four cell lines we used consisted of adenocarcinoma and squamous carcinoma. Using these four cell lines, we preliminarily depicted a raw landscape of PPIP5K2 function in NSCLC. Studies in the subtype of NSCLC will further enrich the acknowledgement that PPIP5K2 may differentially contribute to the development of NSCLC. Our research demonstrated that the loss of function of PPIP5K2 remarkably inhibited the proliferation and EMT-dependent metastasis of NSCLC both in vitro and in vivo, which is in line with the conclusion derived from PPIP5K2 overexpressed cells.

Mutations in some key genes of the AKT/mTOR pathway, such as PIK3CA, PIK3R1, PTEN, AKT, mTOR, etc., can lead to the abnormal activation of the PI3K-AKT-mTOR pathway, which in turn leads to tumorigenesis [30,31]. We found that the overexpression of PPIP5K2 increased in the phosphorylation levels of AKT, mTOR, 4E-BP1, and s70s6k, whereas PPIP5K2 knockdown led to opposite effects. Furthermore, we confirmed in vitro and in vivo that PPIP5K2 overexpression or knockdown may alter NSCLC proliferation. Given the evidence from a properly established metastatic model plus the current in vitro evidence, the effects of PPIP5K2 on cell invasion and metastasis of USCLC and that AKT signaling pathway agonists or antagonists could reverse these effects elicited by PPIP5K2 silence or overexpression can be fully unraveled.

Up to now, the FDA has already approved the allosteric inhibitors of mTOR, everolimus, and temsirolimus for the treatment of advanced cancers of the kidney, breast, and pancreas [32]. Four PI3K inhibitors have now received regulatory approval for indications involving non-Hodgkin lymphoma or chronic lymphocytic leukemia [33]. In addition, a vast number of small molecule inhibitors that target the PI3K/AKT/mTOR pathway—particularly those that target AKT, the major molecule in this signaling pathway—have been utilized in clinical trials, but failed with lower objective response rate due to substantial side effects and safety concerns [34,35,36,37]. Therefore, it is advantageous to diverge from the previously recognized targets and investigate novel targets within the peripheral branches in order to further our understanding of the biological consequences of PI3K/AKT/mTOR signaling, and benefit from this anti-tumor therapeutic strategy. In the present study, we found that PPIP5K2 can promote NSCLC tumorigenesis through activating AKT/mTOR signaling. Owing to the phosphatase activities, aberrant expression of PPIP5K2 will no doubt significantly impact a vast number of substrates, including the key players in AKT/mTOR signaling cassette [38]. Indeed, in our study, we found that the proliferation and metastasis capacities of NSCLC cells could only be partially recovered when PPIP5K2 silenced cells were treated with AKT/mTOR agonists, while the capacities of PPIP5K2 overexpressed NSCLC cells were completely restored post treatment of AKT/mTOR antagonists, suggesting that multiple signaling pathways, primarily AKT/mTOR signaling, may be involved in PPIP5K2-mediated NSCLC progression. Of interest, PPIP5K2 has a surface-mounted substrate capture site that is a target for drug discovery through the analysis of its structure, which offers an excellent basis for the further screening and development of related inhibitors [39,40]. The above implies that PPIP5K2 might serve as a predictive biomarker for a better selection of patients responsive to AKT/mTOR inhibitors.

## 5. Conclusions

In summary, our results indicate that PPIP5K2 act as a critical oncogene in NSCLC progression by activating the phosphorylation of AKT/mTOR signaling pathway. This research provides an innovative approach for treating tumors, particularly NSCLC, with an emphasis on the AKT/mTOR signaling pathway by targeting PPIP5K2.

## Figures and Tables

**Table 1:** The primer sequences.

Gene Name	Primers (5′-3′)	
PPIP5K2	Forward	AAGCAATGTACGAAAAACAGGC
	Reverse	AAGGCAGACTTTCCAAGCAAT
Snail1	Forward	TCGGAAGCCTAACTACAGCGA
	Reverse	AGATGAGCATTGGCAGCGAG
Slug	Forward	CGAACTGGACACACATACAGTG
	Reverse	CTGAGGATCTCTGGTTGTGGT
Zeb1	Forward	TTACACCTTTGCATACAGAACCC
	Reverse	TTTACGATTACACCCAGACTGC
GAPDH	Forward	CTGGGCTACACTGAGCACC
	Reverse	AAGTGGTCGTTGAGGGCAATG

**Table 2:** The information on antibodies.

Antibody Name	Brand	Catalog Number
Rabbit anti-PPIP5K2	Sigma	Cat#PA5-29340
Rabbit anti-E-cadherin	CST	Cat#3195S
Rabbit anti-N-cadherin	CST	Cat#13116S
Rabbit anti-β-Catenin	CST	Cat#8480S
Rabbit anti-Vimentin	CST	Cat#5741
Rabbit anti-mTOR	CST	Cat#2983
Rabbit anti-Phospho-mTOR (Ser2448)	CST	Cat#5536
Rabbit anti-4E-BP1	CST	Cat#9644
Rabbit anti-Phospho-4E-BP1 (Thr37/46)	CST	Cat#2855
Rabbit anti-p70 S6 Kinase	CST	Cat#34475
Rabbit anti-Phospho-p70 S6 Kinase	CST	Cat#9234
Rabbit anti-Akt	CST	Cat#4691
Rabbit anti-Phospho-Akt (Ser473)	CST	Cat#4060
Rabbit anti-GAPDH	Proteintech	Cat#10494-1-AP
Rabbit anti-Flag	Proteintech	Cat#20543-1-AP

## Data Availability

The datasets used and/or analyzed during the current study are available from the corresponding author on reasonable request.

## Supplementary Materials

The following supporting information can be downloaded at: https://www.mdpi.com/article/10.3390/cancers16030590/s1, Figure S1: The full-length blots/gels.

## Abbreviations

NSCLC	Non-small cell lung cancer
FBS	Fetal bovine serum
PPIP5K2	Diphosphoinositol pentakisphosphate kinase 2
EMT	Epithelial–mesenchymal transition
RT-qPCR	Quantitative reverse transcription polymerase chain reaction
HE staining	Hematoxylin-eosin staining
IHC	Immunohistochemistry
CCK-8	Cell counting kit-8
