# Supplementary figures and images for: PPIP5K2 Facilitates Proliferation and Metastasis of Non-Small Lung Cancer (NSCLC) through AKT Signaling Pathway
Source: Cancers (Basel). 2024 Jan 30;16(3):590. doi: 10.3390/cancers16030590 (PMC10854519; doi:10.3390/cancers16030590)

Fig.2A

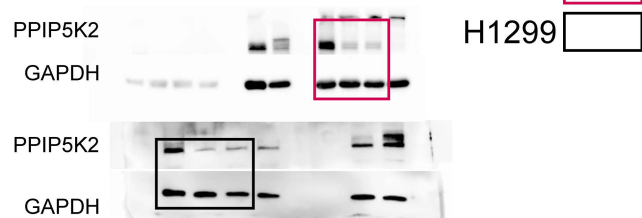

A549 ☐  
H1299 ☐

Fig.2B

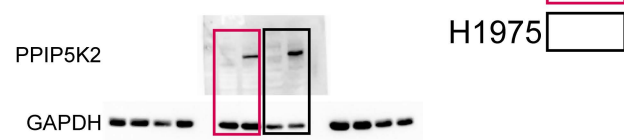

H1703 ☐  
H1975 ☐

Fig.4I

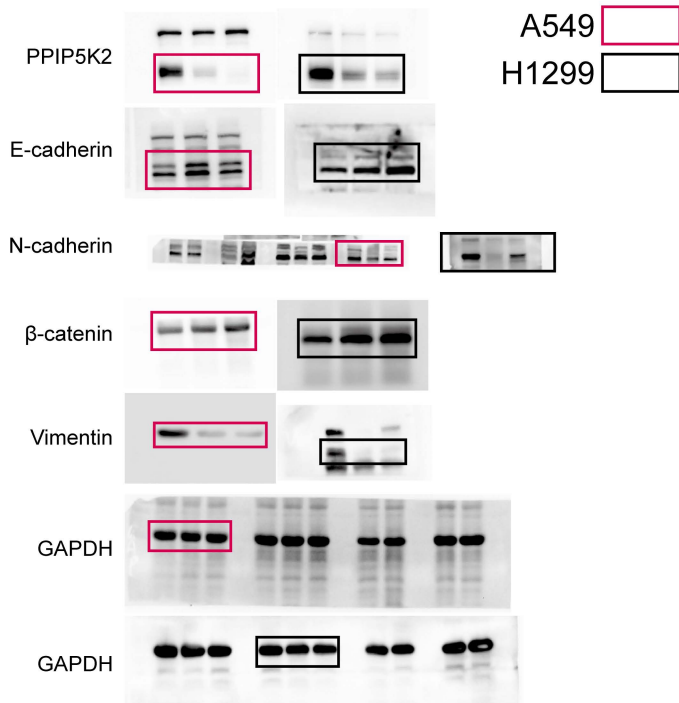

A549 ☐  
H1299 ☐

Fig.4J

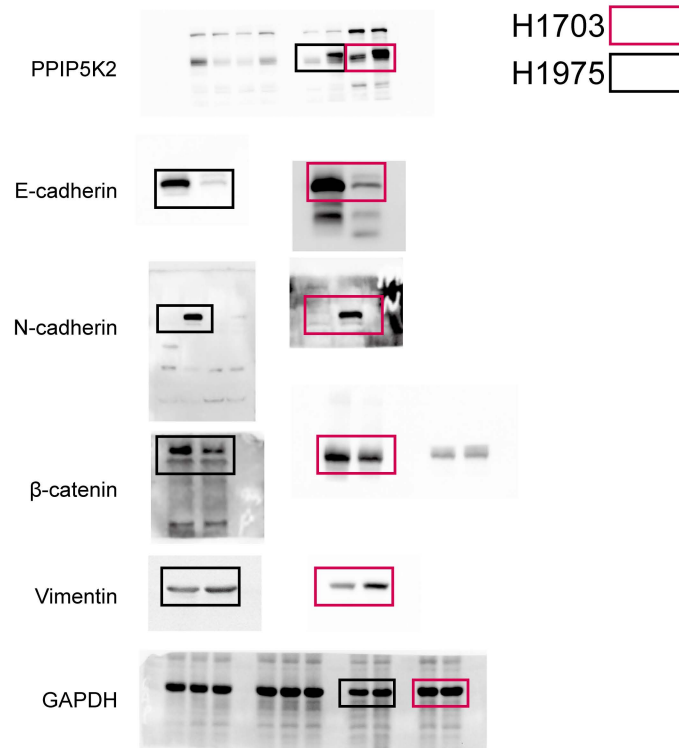

H1703 ☐  
H1975 ☐

Fig.5A

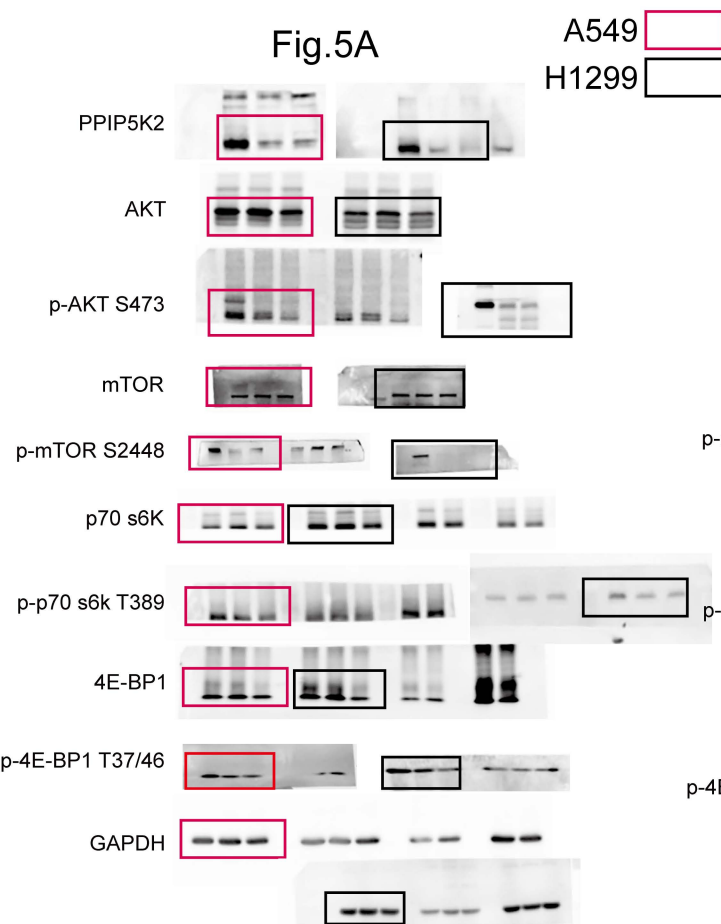

A549 ☐  
H1299 ☐

Fig.5B

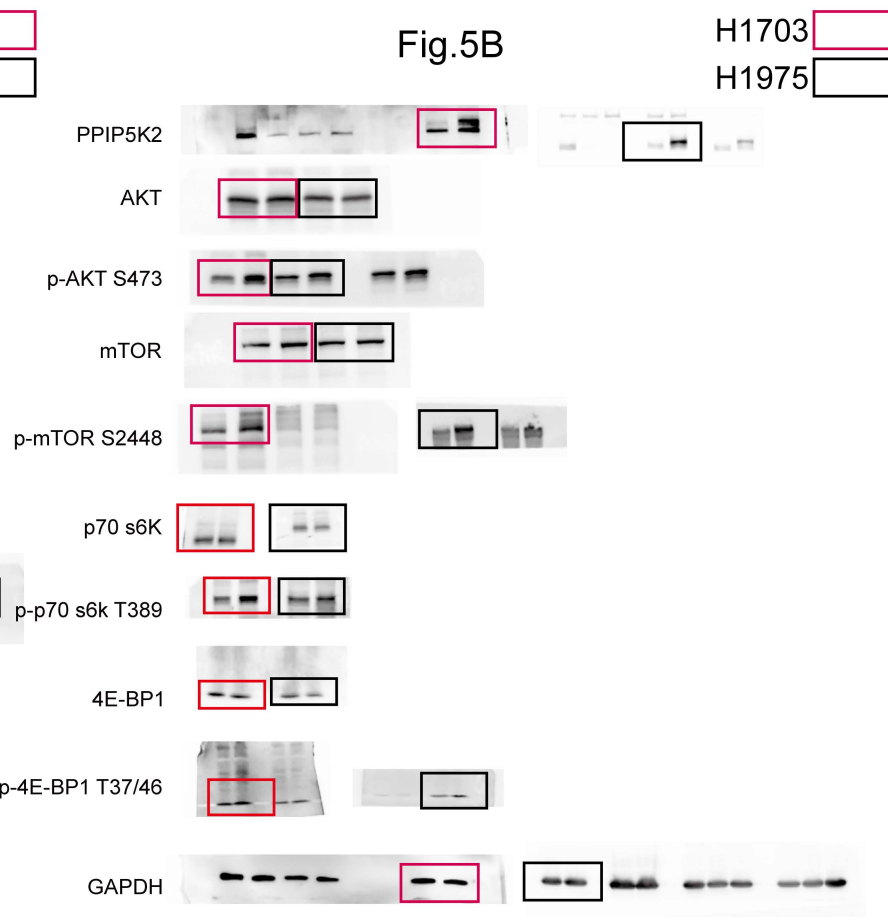

H1703 ☐  
H1975 ☐

Supplement: Supplementary file 1 [file cancers-16-00590-s001.zip › Supplementary Figure S1.pdf]
